# Urinary Bisphenol A Concentration and Angiography-Defined Coronary Artery Stenosis

**DOI:** 10.1371/journal.pone.0043378

**Published:** 2012-08-15

**Authors:** David Melzer, Phil Gates, Nicholas J. Osborn, William E. Henley, Ricardo Cipelli, Anita Young, Cathryn Money, Paul McCormack, Peter Schofield, David Mosedale, David Grainger, Tamara S. Galloway

**Affiliations:** 1 Epidemiology and Public Health Group, Peninsula College of Medicine and Dentistry, University of Exeter, Exeter, United Kingdom; 2 College of Life and Environmental Sciences, University of Exeter, Exeter, United Kingdom; 3 School of Mathematics and Statistics, University of Plymouth, Plymouth, United Kingdom; 4 Brixham Environmental Laboratory, Brixham, United Kingdom; 5 European Centre for Environment and Human Health, Peninsula College of Medicine and Dentistry, University of Exeter, Truro, United Kingdom; 6 Department of Medicine, Cambridge University, Cambridge, United Kingdom; 7 Papworth Hospital NHS Foundation Trust, Cambridge, United Kingdom; California Pacific Medicial Center Research Institute, United States of America

## Abstract

**Background:**

Bisphenol A is widely used in food and drinks packaging. There is evidence of associations between raised urinary bisphenol A (uBPA) and increased incidence of reported cardiovascular diagnoses.

**Methodology/Principal Findings:**

To estimate associations between BPA exposure and angiographically graded coronary atherosclerosis. 591 patients participating in The Metabonomics and Genomics in Coronary Artery Disease (MaGiCAD) study in Cambridgeshire UK, comparing urinary BPA (uBPA) with grades of severity of coronary artery disease (CAD) on angiography. Linear models were adjusted for BMI, occupational social class and diabetes status. Severe (one to three vessel) CAD was present in 385 patients, 86 had intermediate disease (n = 86) and 120 had normal coronary arteries. The (unadjusted) median uBPA concentration was 1.28 ng/mL with normal coronary arteries, and 1.53 ng/mL with severe CAD. Compared to those with normal coronary arteries, uBPA concentration was significantly higher in those with severe CAD (OR per uBPA SD = 5.96 ng/ml OR = 1.43, CI 1.03 to 1.98, p = 0.033), and near significant for intermediate disease (OR = 1.69, CI 0.98 to 2.94, p = 0.061). There was no significant uBPA difference between patients with severe CAD (needing surgery) and the remaining groups combined.

**Conclusions/Significance:**

BPA exposure was higher in those with severe coronary artery stenoses compared to those with no vessel disease. Larger studies are needed to estimate true dose response relationships. The mechanisms underlying the association remain to be established.

## Introduction

Bisphenol A (BPA) is a synthetic monomer used in the manufacture of polycarbonate plastics and in the epoxy resins lining food and beverage containers, and is one of the world’s highest production volume chemicals [1]. BPA is classified as an endocrine disrupting chemical (EDC) due to reported interference in hormonal function and signaling, especially in relation to estrogen receptor-mediated pathways [2]. Ubiquitous human exposure to BPA is evident from the presence of detectable concentrations of BPA metabolites in the urine of 80–90% of the population worldwide [3], [4]. The main source of exposure for humans is not clearly understood, but is most likely through consumption of packaged food and beverages, with additional exposure from drinking water, dental sealants, dermal exposure and inhalation of household dusts [5].

The first major epidemiological analyses of adult health effects associated with exposure to BPA involved a study of 1455 adults aged 18 to 74 years with measured urinary BPA (uBPA) from the US National Health and Nutrition Survey (NHANES) 2003–2004 [6]. Higher BPA concentrations were associated with cardiovascular diagnoses (OR per one SD increase in BPA concentration 1.39, 95% CI 1.18–1.63; p = 0.001 with full adjustment). The association with cardiovascular disease was also present in a separate population representative sample in NHANES 2005/06 (n = 1493), despite a decrease in uBPA concentrations of around 30% compared to the 2003/04 results. Associations with diabetes and some liver enzyme changes did not reach significance in the 2005–2006 data, but remained significant in pooled data [7]. This cross-sectional data has recently been augmented by a longitudinal study, in which higher uBPA concentrations were shown to predict first diagnoses of coronary artery disease (CAD) during a 6 year follow-up in the European Prospective Investigation of Cancer EPIC-Norfolk study, greatly strengthening the evidence for a causal role for BPA in the disease process [8]. In this prospective association the uBPA concentrations were relatively low, with median values of 1.35 ng/mL in the EPIC-Norfolk cases and 1.24 ng/mL in the controls, compared to a median value of 2.6 ng/ml (interquartile range 1.3 to 5.6) in the original NHANES study of 2003/4 [8], [9].

The mechanism by which exposure to BPA affects CAD incidence is unknown. Our sensitivity analyses showed that the association between uBPA and incident CAD appears to be independent of classical CAD risk factors [8]. In particular, exclusion of those with obesity and adjustment for blood lipid concentrations and levels of physical activity had little effect, suggesting that obesity related risks and incidental higher BPA ingestion (and hence excretion) are an unlikely explanation for the findings.

Previous studies have involved self-reported diagnoses, whilst incident disease was identified from recorded hospital admission or death certification. In the current study, we tested the hypothesis that greater exposure to BPA would be associated with coronary atherosclerosis, to gain a clearer understanding of the nature and specificity of the association between BPA exposure and CAD. We studied a population with vessel disease precisely defined at angiography, from The Metabonomics and Genomics in Coronary Artery Disease (MaGiCAD) study, an angiography referral study from East Anglia, UK. This patient population is drawn from the EPIC-Norfolk and neighbouring geographical areas, referred to the regional angiography centre at Papworth Hospital.

## Methods

The MaGiCAD study was designed to study risk factors for coronary heart disease in the UK, and was carried out at Papworth Hospital NHS Trust, Cambridgeshire UK, a regional cardiac centre [10]. All patients attending for a diagnostic angiogram were eligible, excluding those who had previously had a heart transplant or were unable to give informed consent: 1336 patients agreed to participate. For logistic reasons, patients undergoing procedures between 10∶00 and 14∶30 were included (to allow processing of blood, urine specimens etc). In a comparison of those patients recruited against those not recruited, there were no significant differences in disease severity (assessed using angiogram). All of the patients attending Papworth Hospital for an angiogram must have a suspected cardiac disorder because of the risk associated with the diagnostic procedure. Nevertheless, 19.2% (n = 235 of 1,221 with angiography results) of these patients had normal coronary arteries. The sub-group of patients with angiographically normal coronary arteries, despite their apparent lack of coronary atherosclerosis, may have another cardiac disorder or suspected cardiac disorder (e.g. a diseased aortic valve or non-attributable chest pain).

For the current analysis, we included the 591 patients who provided a useable urine specimen during their angiography visit and who were undergoing first angiographies: urine samples were collected between 28 November 2001 and 9 November 2004 and stored at −80°C.

### Classification of Vessel Disease

The index used to classify coronary disease severity was a minor modification of that described by Ringqvist [11], with scores ranging from normal coronary arteries to disease in three vessels. The right coronary artery, left anterior descending artery, and left circumflex artery were considered diseased if there was a ≥70% reduction in the internal diameter of the vessel or major subvessels (Table 1). The left main stem coronary artery was considered diseased if there was a ≥50% reduction in diameter. The number of vessels diseased was calculated according to right or left dominance of the coronary circulation, as summarised in Table 1. Participants who had evidence of disease but did not reach the criteria outlined above were classified as having disease of intermediate severity for the purpose of this study. Participants with no evidence of disease were classified as having normal coronary arteries.

**Table 1:** Summary of disease scoring method for reading the MaGICAD study angiograms.

Right Dominant Coronary Circulation		Left Dominant Coronary Circulation	
*Diseased vessel	Score	*Diseased vessel	Score
Proximal, mid or distal right coronary artery	1 VD	Left main coronary artery	3 VD
		*If no left main coronary artery disease:*	
Left main coronary artery	2 VD	Proximal, mid or distal left anterior descending or 1^st^ or2^nd^ diagonal artery	1 VD
*If no left main coronary artery disease:*		Proximal, mid or distal left circumflex artery	2 VD
Proximal, mid or distal left anterior descending or 1^st^ or 2^nd^diagonal artery	1 VD	*If no proximal, mid or distal left circumflex* *artery disease:*	
Proximal, mid or distal left circumflex or one obtuse marginalartery	1 VD	Any one of three obtuse marginal	1 VD
		Left atrio-ventricular artery, 1^st^ –3^rd^ posterolateral or left posterior descending artery	1 VD

* Note that vessels are considered diseased according to criteria outlined in the text.

To avoid possible bias of interpretation by the original clinician, the angiogram of every patient in the study was re-read. The video file was extracted from the hospital database and the anonymised angiogram was read by two clinicians and a native vessel disease status calculated. Between each of the independent reads and the result of the angiogram read for clinical purposes there was approximately 70% identity (disease status the same), and 95% similarity (disease statuses within one category of each other).

### Analysis of Urinary BPA Concentrations

Analysis of uBPA metabolites in the spot urine specimens was performed in 2010/11 by Brixham Environmental Laboratory, Division of Analytical Chemistry (a division of AstraZeneca PLC) in compliance with Good Laboratory Practice, EU Directive 88/32/EEC. Because orally administered BPA is considered to be rapidly and completely excreted, and is stable in frozen urine, urine is the body fluid most appropriate for biomonitoring assessment of BPA exposure and we followed WHO guidelines over study design to evaluate exposure [12]. A comprehensive, GLP compliant quality control procedure was followed including reagent blanks and measurement of both free and conjugated BPA, to confirm that there was minimal leaching of BPA from vessel walls. Total (free and conjugated) urinary concentrations of BPA were obtained using online, solid-phase extraction (SPE) coupled with high performance liquid chromatography (LC)-isotope dilution tandem mass spectrometry (MS/MS) with peak focusing [13], based on the methods employed by NHANES and adopted by the Division of Environmental Health Laboratory Sciences, National Centre for Environmental Health, Centre for Disease Prevention,(CDC) [3]. Calibration was linear from 0.50–100 µg/L (R^2^>0.996), limit of detection was <0.50 ng/ml uBPA, limit of quantification, 0.50 ng/ml uBPA, lowest calibration standard gave a signal height:noise ratio >10 (relative standard deviations <20%, all other standards <15%).

### Statistical Analysis

As previously [13] our lower level of accurate BPA detection (LLOD) was <0.5 ng/ml, below which values of 0.28 were assigned, following the methods of Calafat and colleagues [3] (117 cases reassigned). Logistic regression models were used to estimate odds ratios for CAD status as outcome with uBPA concentrations (standardized z-scores) and covariates as explanators. Adjustment was for: age, sex, occupational social class (grouped into uncoded, professional, managerial, skilled non-manual, skilled manual, semiskilled, non-skilled) and body mass index (BMI, measured weight in kilograms divided by the square of measured height in meters, categorized into: underweight (BMI<18.5), recommended (BMI 18.5 to 24.9), overweight (BMI 25.0 to 29.9), obese I (BMI 30.0 to 34.9), obese II (BMI 35.0 or above). Sensitivity analyses included adjustment for smoking (pack years) and alcohol intake (estimated units per week). Regression models were adjusted for an available occupationally based social class measure, as Calafat and colleagues [9] reported that socio-economic status is associated with BPA concentrations.

A measure of urine concentration was not available, so we have provided a sensitivity analysis adjusted for blood urea creatinine ratio, a validated clinical measure of hydration [14].

## Results

There were 591 patients with first angiograms and uBPA values: one or more severe stenoses were present in 385 (65.1%) patients, and 120 patients had normal coronary arteries (20.3%). The remainder of patients had one or more abnormalities in their coronary arteries, but did not classify as 1-, 2- or 3-VD when applying the categorisation method used; these 86 patients (14.6%) were classified as having intermediate disease. Overall, 32% of the sample was female, but this percentage was 50% and 51.7% in the intermediate disease and normal coronary artery groups respectively. Those with no stenoses tended to be in the younger age groups. There was no difference in BMI categories between the groups or in occupational social class, but those with intermediate stenoses were less likely to have diabetes.

Bisphenol A concentrations varied from below levels of detection (n = 117, 16.9%) to 69.4 ng/mL. As in previous biomonitoring studies, the distribution was strongly skewed with most values being relatively low (Table 2). The (unadjusted) median uBPA value was 1.28 ng/mL in those patients with normal coronary arteries, and 1.53 ng/mL in those with severe disease. Median uBPA concentration in the relatively small intermediate group (n = 86, median 1.58 ng/ml) was higher than in those with severe disease. Overall 17.5% of patients with normal coronary arteries had uBPA concentrations ≥3 ng/mL compared to 30.2% in the intermediate group and 26.7% in the severe group.

**Table 2:** Characteristics of the sample by severity of coronary artery stenoses.

	None		Intermediate		three vessel		Total n %		p-value
	N	%	n	%	n	%	n	%	
*Total*	120	(100)	86	(100)	385	(100)	591	(100)	
Gender									<0.001
Female	62	(51.7)	43	(50)	84	(21.8)	189	(32)	
Male	58	(48.3)	43	(50)	301	(78.2)	402	(68)	
Age-group (years)									0.002
30 to 45	12	(10)	6	(7)	19	(4.9)	37	(6.3)	
46 to 64	74	(61.7)	37	(43)	170	(44.2)	281	(47.6)	
64 to 84	33	(27.5)	42	(48.8)	189	(49.1)	264	(44.7)	
85 to 95	1	(0.8)	1	(1.2)	7	(1.8)	9	(1.5)	
BMI category									0.93
≤18.5	1	(0.8)	0	(0)	1	(0.3)	2	(0.3)	
18.5 to 25	32	(26.7)	24	(27.9)	112	(29.1)	168	(28.4)	
>25 to 30	52	(43.3)	37	(43)	178	(46.2)	267	(45.2)	
>30	31	(25.8)	23	(26.7)	85	(22.1)	139	(23.5)	
Unknown	4	(3.3)	2	(2.3)	9	(2.3)	15	(2.5)	
Diabetes									
No	111	(92.5)	74	(86.1)	309	(80.3)	494	(83.6)	0.004
Present	5	(4.2)	11	(12.8)	69	(17.9)	85	(14.4)	
Unknown	4	(3.3)	1	(1.2)	7	(1.8)	12	(2)	
Occupational social class		8		9		20		37	0.111
Unknown	8	(6.7)	9	(10.5)	20	(5.2)	37	(6.3)	
Professional	4	(3.3)	2	(2.3)	21	(5.5)	27	(4.6)	
Managerial	29	(24.2)	20	(23.3)	102	(26.5)	151	(25.6)	
Skilled non-manual	26	(21.7)	20	(23.3)	103	(26.8)	149	(25.2)	
Semiskilled non-manual	37	(30.8)	14	(16.3)	70	(18.2)	121	(20.5)	
Skilled manual	12	(10)	16	(18.6)	51	(13.3)	79	(13.4)	
Non-skilled manual	4	(3.3)	5	(5.8)	18	(4.7)	27	(4.6)	
Urinary Bisphenol A concentration (ng/mL)									
Mean (Standard deviation)	2.13	(2.73)	3.31	(7.37)	3.82	(6.31)	3.14	(5.96)	
Median	1.28		1.77		1.53		1.53		
Inter-quartile range	0.69 to 2.26		0.92 to 3.73		0.78 to 3.26		0.78 to 3.03		

The formal classification of angiography results for clinical purposes was at the time of assessment primarily into patients with one or more severe stenoses versus the rest: uBPA was not associated with severe disease in this dichotomy in age and sex adjusted models (per uBPA standard deviation  = 5.96 nm/ml, OR = 1.14 CI 0.94 to 1.38, p = 0.172) or fully adjusted models (OR = 1.11 CI 0.93 to 1.33, p = 0.24).

On separating those with normal coronary arteries from those with intermediate severities of disease, in models adjusted for age and sex only (Table 3), uBPA concentrations were significantly higher in those with severe CAD (versus no CAD: per uBPA standard deviation odds ratio was 1.51 95% CI 1.07 to 2.14, p = 0.019). Comparison of the smaller, intermediate CAD category to those with normal coronary arteries narrowly missed significance (OR = 1.79 CI 0.98 to 3.27, p = 0.058).

**Table 3:** Odds ratios per SD increase in uBPA (5.96 ng/ml) of diagnosed coronary artery features comparing age and sex adjusted models, fully adjusted models and fully adjusted models where the severity of disease is further broken down to show 1, 2, and 3 vessel disease (VD).

	Number	Odds ratioper SD increase	95%CI	p-value
Age and sex adjusted				
None	120	1		
Intermediate	86	1.79	(0.98 to 3.27)	0.058
Severe	385	1.51	(1.07 to 2.14)	0.019
Fully adjusted models				
None	120	1		
Intermediate	86	1.69	(0.98 to 2.94)	0.061
Severe	385	1.43	(1.03 to 1.98)	0.033
Fully adjusted detailed models				
None	120	1		
Intermediate	86	1.69	(0.98 to 2.94)	0.061
1 VD	148	1.42	(1.02 to 1.98)	0.036
2 VD	123	1.20	(0.70 to 2.04)	0.501
3 VD	114	2.09	(1.62 to 3.46)	0.004

Note: full adjustment included age, sex, BMI category, occupational social class and diabetes status.

In models comparing patients with severe CAD with patients with normal coronary arteries additionally adjusted for occupational social position, BMI category and diabetes status (Table 3), the estimate was only a little changed: the OR per SD of uBPA comparing patients with severe disease and no disease was 1.43 (CI 1.03 to 1.98, p = 0.033). Although numbers are small, we also present comparisons between uBPA in patients with one, two and three vessel disease versus those with no disease. There were significant differences between patients with one vessel disease versus none (OR = 1.69 CI 1.02 to 1.98, p = 0.036), but the comparison between patients with two vessel disease and those with normal coronary arteries was not significant. The largest association found was comparing patients with three vessel disease versus patients with no disease (OR = 2.09 CI 1.62 to 3.46, p = 0.004).

### Sensitivity Analyses

We conducted post-hoc sensitivity analyses to check the robustness of the association of higher uBPA comparing severe to no vessel disease. Firstly we adjusted the ‘full’ model comparing severe CAD to the normal artery group additionally for serum creatinine (to account for renal functioning) and alcohol intake (in weekly standard units of alcohol per week): the result was little changed (OR 1.42 CI 1.01 to 1.99, p = 0.043). Secondly we excluded those aged 75 and over, to avoid possible multiple morbidity: this had little effect (OR = 1.44 CI 1.02 to 2.03, p = 0.038). Formal tests for interaction between uBPA and age (p = 0.241) or diabetes status (p = 0.508) were non-significant.

As urine BPA concentrations might be affected by the patient’s state of hydration we additionally adjusted estimates for the ratio of blood urea nitrogen and blood creatinine concentrations: the results of the fully adjusted models as in Table 3 were only marginally different: intermediate vs none OR = 1.70 (CI 0.98 to 2.94, p = 0.060) and severe vessel disease OR = 1.51 (CI 1.07 to 2.14, p = 0.020).

## Discussion

In this study, we aimed to assess whether increased uBPA was associated with angiography defined coronary artery stenoses (coronary atherosclerosis). We studied a relatively small sample from those undergoing first angiography in a regional cardiac centre from 2001 to 2004. The centre in question serves the EPIC Norfolk cohort study population in which we have previously shown that higher uBPA concentration was predictive of incident cardiovascular disease [8]. In EPIC-Norfolk, diagnoses were clinically confirmed from hospital episode data and mortality records. In the current study, we found evidence of higher uBPA concentrations in those with intermediate or severe stenoses compared to those graded as having no coronary artery disease, in independently read angiograms.

These results are important as they suggest that associations between uBPA and CAD may be specific to coronary artery stenosis. Associations between uBPA and cardiovascular disease have now been reported in three previous studies, in which disease diagnosis data was drawn from questionnaire self-report [6] or identified from clinical notes [8]. In NHANES 2003/2004 and again in NHANES 2005/2006, higher uBPA concentrations were associated with heart disease (pooled p value <0.001). A major limitation of these studies is their cross-sectional nature, leaving open the possibility of reverse-causation, e.g. that patients with CAD may have changed their behaviour or diet in a way that incidentally led to increased exposure to BPA. A prospective design was therefore adopted with 758 incident CAD cases and 861 controls from the European Prospective Investigation of Cancer (EPIC-Norfolk), UK study, which concluded that higher uBPA concentrations (>4 ng/mL) were associated with incident CAD (OR 1.39 95% CI 1.06 to 1.84, p = 0.020). This longitudinal study effectively ruled out reverse causation, strengthening the evidence for causal inference.

A cross-sectional analysis of pooled data from NHANES 2003–2006 showed that uBPA was associated with general and central obesity [15], confirming earlier findings of an association between daily excreted BPA and waist circumference in Italian men [13]. However, sensitivity analyses of the 2011 EPIC study excluded those with obesity and adjusted for blood lipid concentrations and levels of physical activity, yet found little effect on the association; neither did adjustment for vitamin C as a marker of diets poor in fruit and vegetables, or adjustment for education, smoking or blood pressure. The uBPA association with CAD incidence in the EPIC sample therefore appears to have been independent of conventional CAD risk factors. This conclusion was also reached in a study of 1016 elderly subjects [16] which showed a weak relationship between circulating BPA and the thickness and composition of the intima-media complex in the carotid artery, considered to be a marker of lipid infiltration of the vascular wall [17]. The effect remained significant after adjustment for conventional CAD risk factors, suggesting that any vascular effect was occurring independently of these.

Our analysis shows that BPA was similarly elevated in patients with intermediate vessel changes and patients with severe stenoses (combining the individually small groups with 1-, 2- and 3- vessel disease). This is consistent with observations that coronary plaques develop focally, independently, and exhibit morphologic and histological heterogeneity between and within coronary arteries [18]–[20]. Localization of coronary lesions occurs despite similar exposure to pro-atherosclerotic risk factors and this has been attributed to localized hemodynamic disturbances, especially to endothelial shear stress [18], [21]. Endothelial shear stress promotes an athero-protective endothelial cell phenotype, but low endothelial shear stress induces an atherogenic phenotype that reduces nitric oxide bioavailability, promotes low density lipoprotein cholesterol uptake and oxidation, recruits inflammatory cells and promotes smooth muscle cell migration to the intima [18], [19]. Localized areas of low endothelial shear stress are found at branch points, bifurcations and areas of large curvature in coronary arteries. These regional differences in hemodynamics appear to prime endothelial cells to respond distinctly to systemic risk factors [22]. If BPA plays a direct role in promoting atherosclerosis, our observations of individual differences in plaque burden in the presence of similar BPA exposure probably reflects the heterogenic susceptibility of specific vessels, and specific regions within vessels, to atherogenesis and plaque progression.

The mechanism by which BPA ingestion and metabolism influences vascular function and risk of cardiovascular disease has not been elaborated. A number of environmental contaminants have been associated with vascular endothelial dysfunction [23], [24] and accelerated progression of atherosclerosis [25], [26]. We recently suggested plausible mechanisms by which BPA might increase the risk of cardiovascular disease [7], including reduced nitric oxide bioavailability, altered vascular reactivity to endothelin-1, oxidative stress and inflammation. Laboratory exposure studies have shown that BPA can induce oxidative cellular damage in a range of experimental contexts [27]–[29]. For example, oral intake of BPA over a 30 day period led to oxidative stress in rat hepatocytes. Circulation of BPA and redistribution around the body could contribute to oxidative endothelial cell damage, a suggestion supported by a reported positive associations between urinary BPA and oxidative stress markers (malondialdehyde and 8-deoxyguanosine) in a study of 960 adults [30]. BPA shows estrogen and anti-androgen activity [31] hence BPA may exert estrogenic effects or antagonize endogenous estrogens in cardiovascular tissues by binding to soluble or membrane bound estrogen receptors [32]. We recently reported associations between higher uBPA concentrations and higher estrogen receptor beta, (ERβ) expression and estrogen-related receptor alpha (ERRα) expression [33], reinforcing the evidence for estrogenic activity *in vivo*.

BPA is also capable of causing non-genomic effects in vitro. A rapid disruption of Ca^2+^ homeostasis has been shown in a range of cell types [34] suggesting the involvement of cellular transport mechanisms and ion channels. Estrogen and estrogen-receptor modulators can increase the activity of large conductance Ca ^2+^/voltage-sensitive K^+^ (Maxi-K; K_Ca_ 1.1) channels [35]. Recently, BPA in the micromolar range was shown to activate Maxi-K (K_Ca_1.1) ion channels in human coronary smooth muscle cells in culture, sufficient to hyperpolarise the membrane potential [36]. The effects of BPA on channel activity were rapid (<1 min) and reversible and could theoretically lead to a decrease in excitability in cells that express other voltage dependent ion channels.

### Limitations

One limitation of this study is that the BPA measures are from single spot urine samples, which could be considered as limited measures of long term exposure. Urine is however the recommended matrix of choice for biomonitoring studies of BPA, since BPA is rapidly metabolized and excreted from the body and remains stable in frozen urine on long term storage [3]. Single spot samples have been found to be moderately sensitive in predicting an individual’s longer term tertiary BPA categorization [37], [38] measured stability of BPA over 2 week intervals in first voided urine samples from 60 women and found a Spearman correlation of 0.5, indicating that around 75% of the variability of BPA was unaccounted for. Exposure of the general population to BPA is considered to be primarily from food and beverage consumption and a recent study has highlighted the inhibitory effect of food on first pass metabolism, which would lead to a longer residence time for BPA in the body [39]. There is in addition considerable evidence for non-food exposure to BPA. For example, Zalko and colleagues [40] show that viable skin efficiently absorbs and metabolises BPA, which would bypass first pass metabolism. Given that BPA is widely found in receipt papers, from where transfer to skin can occur [41], frequent exposure of the general population to BPA through this non-food source is a potential additional route of exposure.

This could help to explain why despite its rapid metabolism, BPA is present in such a large percentage of the population at any one time. It is likely that the use of single spot samples would, if anything, result in a smaller (diluted) estimate of the strength of association between BPA and CAD.

Another major limitation is our relatively small sample sizes. Small sample size (n = 86 for the intermediate group) may explain why the central estimate for the uBPA intermediate vs none vessel disease estimate is larger than that for the severe vessel disease group: the very wide confidence intervals on this intermediate estimate (CI 0.98 to 3.27, Table 3) makes interpretation of this hazardous and argues for a better powered study. Because of the temporal variability in BPA exposure and errors in estimation by urine specimens, larger sample sizes are needed. Although our strongest association was found on comparing three vessel disease to none, the intermediate uBPA associations across increasing severity groups show no obvious dose-response relationship. This may be due to possible mechanisms of effect, but may well also be influenced by the very small sample sizes in each specific severity group. Larger studies are clearly needed to estimate the dose response curve for the detailed groupings. It is noteworthy that Ning et al [42] did not find a monotonic association between BPA and the risk of the presence of self-reported diabetes, despite clear statistical differences across quartiles of exposure.

Much remains unknown about the mechanisms involved in the association between BPA and CAD in humans. Future scientific work in humans is, of course, constrained by ethical limits and the practicality of repeated BPA exposure measures and long term follow-up studies. Without these constraints, controlled trials would be needed to prove causation in humans, but such evidence is almost certainly beyond reach.

### Conclusions

In our relatively small sample of patients investigated for ischemic heart disease referred for coronary angiography, BPA exposure (evident in urinary BPA concentrations) was higher in those with severe coronary artery stenoses compared to those with no vessel disease. Larger studies are needed to estimate true dose response relationships. The mechanisms underlying the association remain to be established.
