# Correction: Urinary Bisphenol A Concentration and Angiography-Defined Coronary Artery Stenosis

**DOI:** 10.1371/annotation/5f293018-48a3-40ae-96b7-04438d1d9cb9

**Published:** 2012-11-09

**Authors:** David Melzer, Phil Gates, Nicholas J. Osborn, William E. Henley, Ricardo Cipelli, Anita Young, Cathryn Money, Paul McCormack, Peter Schofield, David Mosedale, David Grainger, Tamara S. Galloway

The third author's name was spelled incorrectly. The correct name is: Nicholas J. Osborne.

The correct citation is: Melzer D, Gates P, Osborne NJ, Henley WE, Cipelli R, et al. (2012) Urinary Bisphenol A Concentration and Angiography-Defined Coronary Artery Stenosis. PLoS ONE 7(8): e43378. doi:10.1371/journal.pone.0043378 

